# Allele Frequency Matching Between SNPs Reveals an Excess of Linkage Disequilibrium in Genic Regions of the Human Genome

**DOI:** 10.1371/journal.pgen.0020142

**Published:** 2006-09-08

**Authors:** Michael A Eberle, Mark J Rieder, Leonid Kruglyak, Deborah A Nickerson

**Affiliations:** 1 Department of Genome Sciences, University of Washington, Seattle, Washington, United States of America; 2 Lewis-Sigler Institute for Integrative Genomics, Princeton University, Princeton, New Jersey, United States of America; 3 Department of Ecology and Evolutionary Biology, Princeton University, Princeton, New Jersey, United States of America; The Jackson Laboratory, United States of America

## Abstract

Significant interest has emerged in mapping genetic susceptibility for complex traits through whole-genome association studies. These studies rely on the extent of association, i.e., linkage disequilibrium (LD), between single nucleotide polymorphisms (SNPs) across the human genome. LD describes the nonrandom association between SNP pairs and can be used as a metric when designing maximally informative panels of SNPs for association studies in human populations. Using data from the 1.58 million SNPs genotyped by Perlegen, we explored the allele frequency dependence of the LD statistic *r* ^2^ both empirically and theoretically. We show that average *r* ^2^ values between SNPs unmatched for allele frequency are always limited to much less than 1 (theoretical 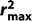 approximately 0.46 to 0.57 for this dataset). Frequency matching of SNP pairs provides a more sensitive measure for assessing the average decay of LD and generates average *r* ^2^ values across nearly the entire informative range (from 0 to 0.89 through 0.95). Additionally, we analyzed the extent of perfect LD (*r* ^2^ = 1.0) using frequency-matched SNPs and found significant differences in the extent of LD in genic regions versus intergenic regions. The SNP pairs exhibiting perfect LD showed a significant bias for derived, nonancestral alleles, providing evidence for positive natural selection in the human genome.

## Synopsis

One of the primary goals for geneticists is isolating regions of the genome that convey increased risk of disease through the association of genetic polymorphisms with phenotypic traits. The recent availability of genome-wide polymorphism data (i.e., single nucleotide polymorphisms [SNPs]) has made association studies possible on an unprecedented scale, and the characterization and selection of these polymorphisms for these studies has been a topic of major interest. One method for choosing informative SNPs has been to compare the correlation between SNPs (a term called linkage disequilibrium), but this can create confounding problems when comparing SNPs of different frequencies. In this study, the authors show that if SNPs are compared to other SNPs of equal or near equal frequency, the correlation between them more accurately represents the true correlation. This also produces a more sensitive method for determining linkage disequilibrium. Using this method, SNPs were compared both within and outside of gene regions to examine the overall correlation between SNPs in each region. Matching SNPs according to their frequency greatly increased the maximum possible correlation and showed significantly higher correlations between SNPs within genes (intragenic) versus between genes (intergenic). Using the recently completed chimpanzee sequence, a larger fraction of high frequency human specific SNPs was found within the perfectly correlated SNP pairs in genic regions compared to intergenic regions. These observations suggest that regions of the genome around genes have been under selective pressure, leading to a greater correlation between SNPs. Genes found in regions with the highest correlations between SNPs will be of particular interest for future genotype-phenotype association studies.

## Introduction

The identification of more than 10 million single nucleotide polymorphisms (SNPs) in the National Center for Biotechnology Institute single nucleotide polymorphism database dbSNP (build 124) provides an extensive database for human genetic analysis. In addition to information on the genomic location of these SNPs, dbSNP also contains individual genotype information for over 2.7 million SNPs. Of these SNPs, 1.58 million have been genotyped on a consistent set of samples by Perlegen in 24 unrelated individuals of European descent, 24 of Han Chinese descent, and 23 of African-American descent [1]. Other large sample sets exist with full genotyping data, such as the approximately 1.1 million SNP genotypes generated in Phase 1 of the International HapMap Project in 30 parent-child trios of European and 30 of African descent and 45 and 44 unrelated individuals of Chinese and Japanese descent, respectively [2]. These genome-wide SNP datasets provide a resource for analyzing genome-wide linkage disequilibrium (LD) structure when selecting SNPs for association studies [1,3–6].

The identification of risk factors for complex traits, where multiple genetic and/or environmental factors contribute to a phenotype, will require the application of a highly dense map of polymorphic markers across the human genome, coupled with sufficiently large sample sizes to achieve adequate power for association mapping [7,8]. It is estimated that 5 to 7 million common SNPs with minor allele frequencies (MAFs) exceeding 10% are present in the human genome [9]. It is well established that the correlation between SNPs decays with physical distance in the genome, due to recombination events between markers, and also depends on population history, recurrent mutation, the frequencies of the markers under comparison, and other factors [10]. LD (the correlation of genotypes between genetic markers) can be used to quantify this effect. The future of whole genome association studies will rely on LD extending over substantial physical distances to identify a causative marker (or genomic interval) even if it is not directly genotyped in a study [11,12] and to select maximally informative markers and decrease genotyping cost.

In this study, we examined the average decay of LD with physical distance using the measure *r* ^2^. Because all measures of LD show some allele frequency dependence in finite sample sizes [13–17], we explored this affect by limiting pairwise LD calculations between SNPs to restricted allele frequency intervals. We find that allele frequency restriction or matching extends the detection of LD and reveals an excess of LD in genic regions of the human genome. These findings provide evidence that natural selection is acting on a significant fraction of all genes (approximately 3%) in the human genome [18–20].

## Results

### Comparison of Linkage Disequilibrium Using Frequency-Matched SNPs

**Figure 1:**
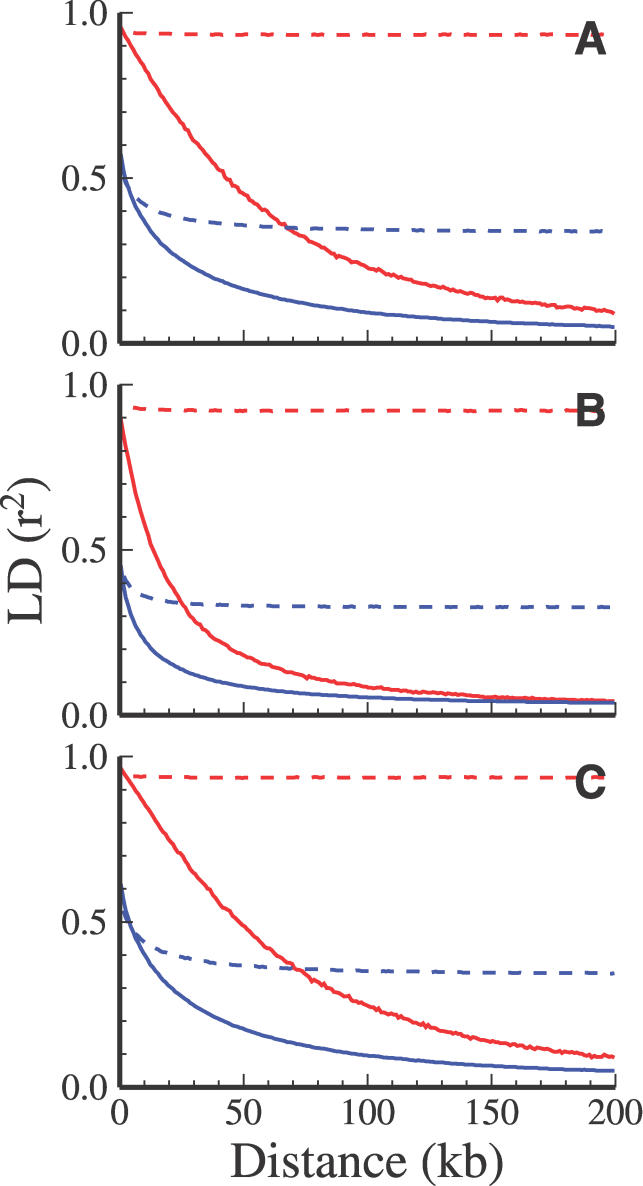
Average Linkage Disequilibrium (*r* ^2^) versus Distance between Markers Linkage disequilibrium (*r* ^2^) in the European (A), African-American (B), and Han Chinese (C) populations. Solid blue lines are average LD values in 1-kb bins excluding SNPs with minor allele frequencies below 0.1, and the dashed blue lines are the theoretical maximum values calculated using the frequency values of all SNP pairs. The red lines are the same except SNP pairs with different minor-allele frequencies were excluded.

Using the 1.58 million SNPs genotyped by Perlegen [1], we explored the impact of allele frequency in calculating genome-wide LD. This dataset was initially selected for analysis because it provided the largest genome-wide genotyped SNP dataset with a consistent ascertainment scheme and minimal data bias [1]. Although a larger, genome-wide SNP dataset (e.g., HapMap, Phase II) has become available, this dataset has a complicated ascertainment scheme that could significantly bias population genetic analysis [21,22]. Therefore, we have focused on the Perlegen dataset, but analysis of HapMap Phase II shows similar findings (unpublished data). For our analysis, we first calculated the average LD using the metric *r* ^2^ (solid blue lines in Figure 1) as a function of distance between pairs of common SNPs (MAF greater than 10%) that were not matched for allele frequency (i.e., with the maximum MAF difference between SNPs extending to 0.4). The LD half-width (distance at which LD decays to 50% of the maximum) was 18, 9, and 19 kb for the European, African-American, and Han Chinese population samples, respectively. These half-widths are probably a slight underestimate of the actual half-widths due to the relatively small sample size, which tends to underestimate the high average *r* ^2^ values at short distances and overestimate the low average *r* ^2^ values at long distances [23].

To test the influence of allele frequency on the *r* ^2^ metric, we recalculated LD between pairs of SNPs matched for allele frequency (Figure 1, red lines). We found that LD half-widths for frequency-matched SNP pairs increased to 40, 12, and 50 kb in the European, African, and Asian samples, respectively. The maximum *r* ^2^ values in the frequency unmatched and matched cases were dramatically different, ranging from 0.45 to 0.62 (unmatched) to 0.90 to 0.97 (matched) and, in both cases, decayed to low *r* ^2^ levels (approximately 0.05) at extended distances (greater than 200 kb). The theoretical maximum *r* ^2^ values (see Materials and Methods) for frequency unmatched and matched SNPs were also dramatically different, ranging from 0.45 to 0.62 (unmatched, dashed blue line, Figure 1) to 0.90 to 0.97 (matched, dashed red line, Figure 1). Average *r* ^2^ values for frequency unmatched and matched cases decayed to low levels (*r* ^2^ ≈ 0.05) at distances greater than 200 kb in all of the population samples.

**Figure 2:**
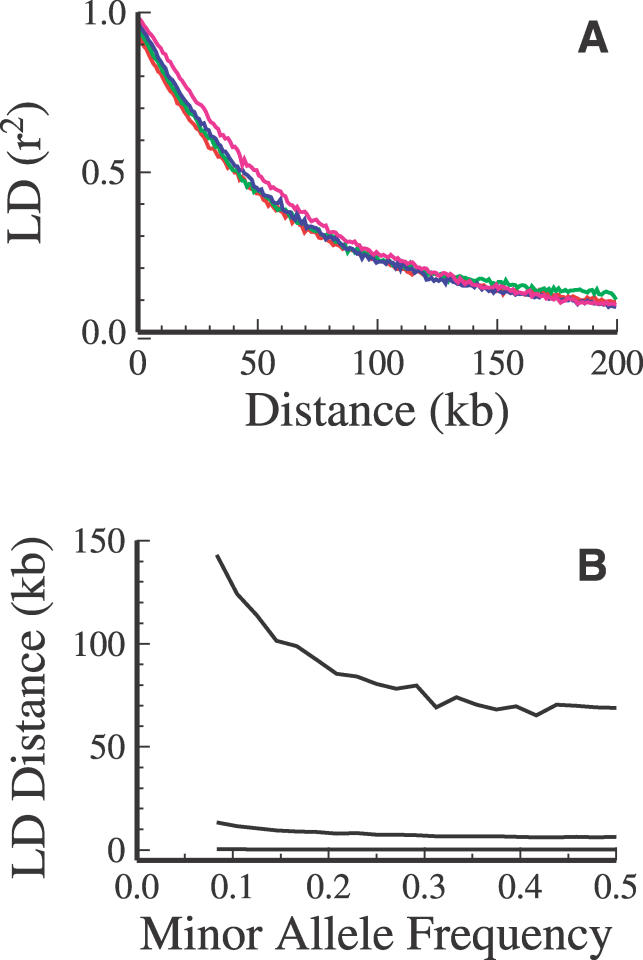
Extent of LD as a Function of Minor Allele Frequency (A) Decay of LD in the European populations for frequency bins 0.1 to 0.2 (red), 0.2 to 0.3 (green), 0.3 to 0.4 (blue), and 0.4 to 0.5 (violet). LD decay curves were calculated using only frequency-matched SNPs (Δf = 0). (B) Distance covered by perfectly correlated SNPs as a function of minor allele frequency in the Europeans. Curves represent the 0.05 (bottom), 0.50 (middle), and 0.95 (top) quantiles of the distributions.

To explore the frequency dependence of r^2^ further, we binned SNPs in 10% MAF intervals (e.g., 10% to 20%, 20% to 30%, etc.) and calculated average LD values for varying physical distances between the SNP pairs. Regardless of the bin, we find that frequency-matched SNPs revealed nearly identical LD decay curves (Figures 2A and S1). This is interesting because it has long been recognized that compared to high-frequency alleles, lower-frequency alleles are usually younger, exhibit less historical recombination, and occur on longer LD blocks [24–26]. To illustrate this, we calculated the physical distance spanned by perfectly correlated SNP pairs across the entire allele-frequency range (Figure 2B). Our analysis shows that less frequent SNPs are indeed more likely to occur in longer blocks. Further analysis of block size showed that lower-frequency SNPs (MAF approximately 8%) had longer average LD blocks (approximately 36 kb) and wider confidence intervals, i.e., 90% confidence interval of approximately 143 kb. LD blocks for high-frequency SNPs (e.g., MAF = 50%) averaged approximately 17 kb and had smaller confidence intervals (90% confidence interval of approximately 68 kb; i.e., 5% of the blocks are shorter and 5% of the blocks are longer). Similar results were observed in the African and Asian samples (Figure S2). This disparity in average LD block size and LD decay is likely due to the overlapping and interleaved nature of the perfectly correlated low-frequency SNPs. Compared to LD blocks for high-frequency SNPs, low-frequency blocks are more likely to overlap (unpublished data), and the correlations between frequency-matched SNPs that reside in different blocks are low.

### LD in Genic and Intergenic Regions

**Figure 3:**
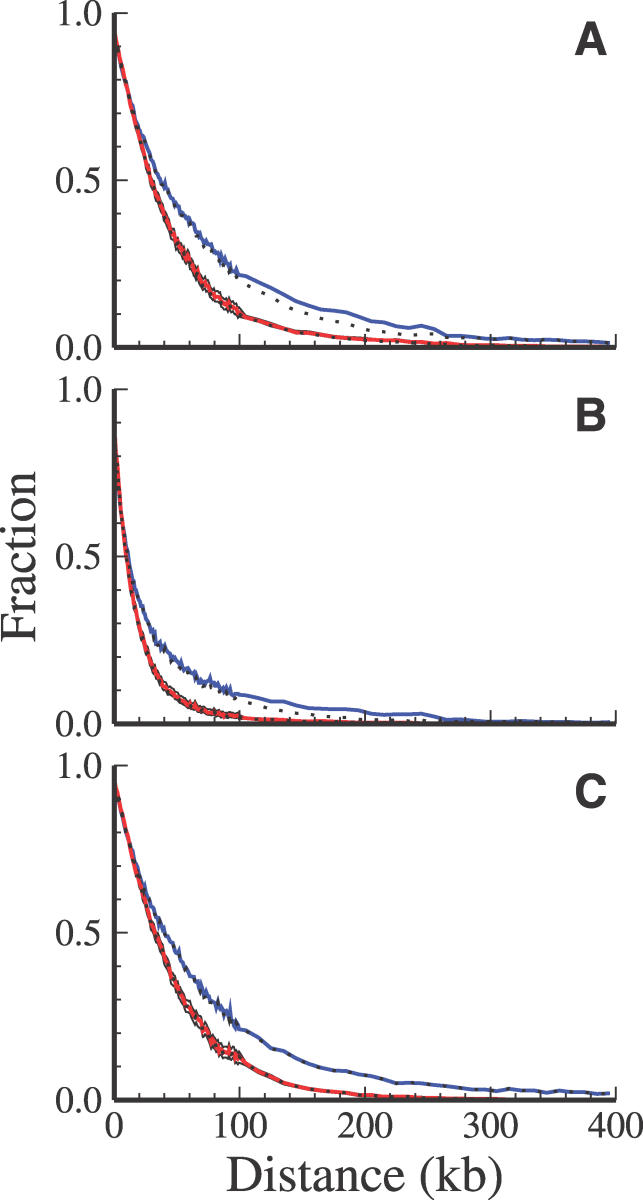
Fraction of SNP Pairs—with Identical Numbers of Minor Allele Observations—in Perfect LD for Intergenic and Genic Regions The dashed lines show the results after removing the 100 regions that contribute the most perfectly correlated SNP pairs for the European (A), African-American (B), and Han Chinese (C) populations.

The observed decay in LD with distance also depends on factors such as population history, selective pressures, mutation, and recombination [10]. Since frequency matching seems more sensitive in detecting LD, we evaluated the decay of LD in genic versus intergenic regions using this approach. Overall, genic regions showed a significantly (*p* << 0.001) larger fraction of perfectly correlated SNPs compared to intergenic regions. Excess LD in genic regions was observed in all populations at physical distances of 20 to 300 kb and extended as far as 400 kb in the European and Han Chinese samples (Figure 3). The spacing between SNPs in genic and intergenic regions is approximately the same, indicating that this difference is not driven by ascertainment bias (see Materials and Methods). This trend was still obvious even after trimming the dataset to eliminate large-scale structural variations, which could introduce LD artifacts (see Materials and Methods). This affect was also not associated with differences in baseline recombination rates between the genic and intergenic regions (Figures S3 and S4).

### Natural Selection and LD

**Figure 4:**
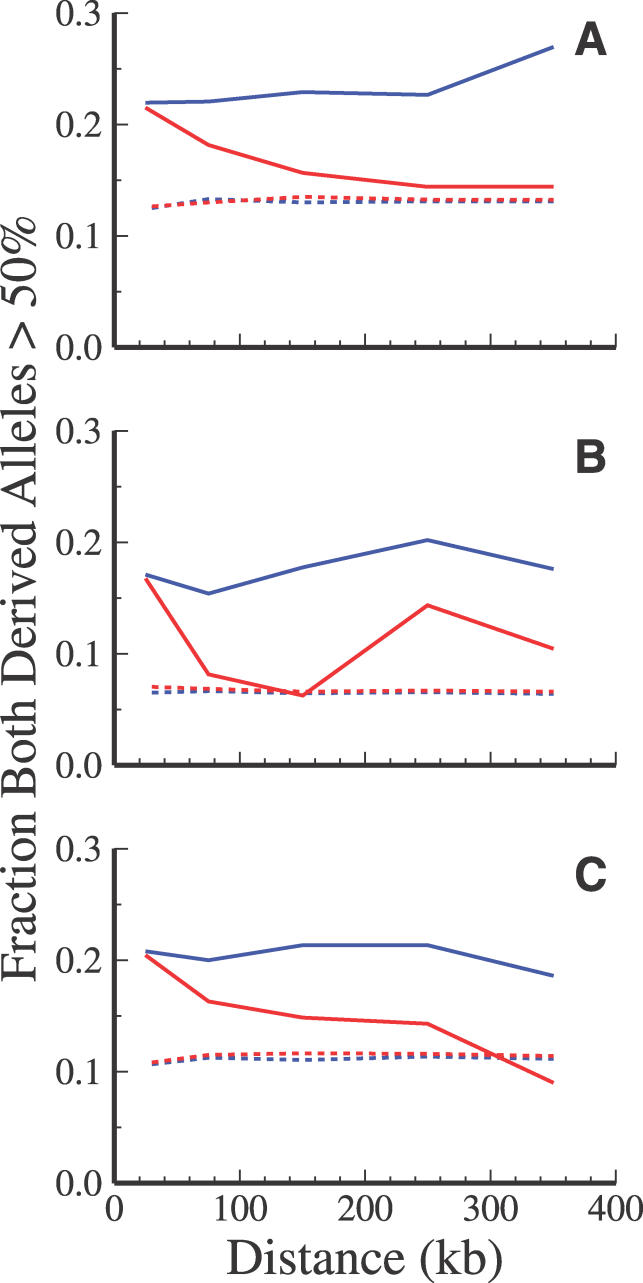
Fraction of SNP Pairs where Both Derived Alleles Occur at Higher Frequencies than the Ancestral Allele Fraction of SNP pairs where both derived alleles occur at higher frequencies than the ancestral allele for European (A), African-American (B), and Chinese (C) populations. Red lines are for intergenic regions and blue lines are for genic regions; solid lines are for perfectly correlated SNP pairs and dashed lines are for SNP pairs with *r* ^2^ < 1.

Since all of the evaluated populations revealed an excess of LD in the genic regions, we then tested for evidence of natural selection. To accomplish this, we determined the origins for perfectly correlated pairs of SNPs, i.e., whether the pairs were ancestral (found in the chimpanzee sequence) versus derived (human specific) in origin. An excess of high-frequency perfectly correlated SNP pairs would be expected when a functional allele is driven upward in frequency by selection. In these instances, nearby SNPs “hitchhike” with the functional SNP and generate a long haplotype with an excess of high-frequency SNPs in strong LD [2,18–20]. Coalescent simulations revealed that compared to the imperfectly correlated SNP pairs (*r* ^2^ < 1), perfectly correlated SNP pairs (*r* ^2^ = 1) should have a lower probability of both SNPs having derived allele frequencies exceeding 50% at distances greater than approximately 80 kb (Figure S5). For imperfectly correlated SNPs, we did not detect a difference in the proportion of SNP pairs having derived alleles exceeding 50% frequency at both positions (7% to 13%) in either genic or intergenic regions. However, perfectly correlated SNPs exhibited a clear excess of high-frequency–derived alleles in both genic and intergenic regions at distances as far as 400 kb. In all populations, the decay of LD was more rapid with physical distance in intergenic regions (Figure 4). This observation is unlikely under the standard neutral model where, for the same recombination distance and population history, older (high-frequency) polymorphisms are expected to occur on smaller haplotype blocks (e.g., Figure 2B) due to a larger number of historical recombinations [27].

**Figure 5:**
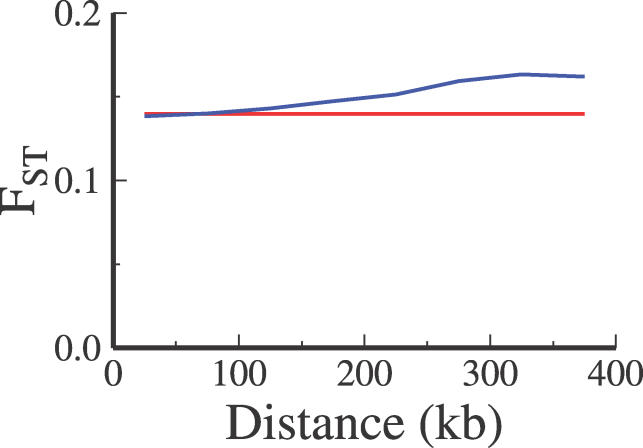
Average Fst Values for the Perfectly Correlated SNPs and All SNPs Red line shows the average values for all frequency matched SNPs according to the distance separating the SNPs in 50-kb bins; Fst values for each SNP are included only once per bin. Blue lines show the corresponding average Fst values for just the perfectly correlated SNPs.

As an additional test for selection, we examined whether perfectly correlated SNPs show systematic frequency bias between populations. For example, SNPs under selective pressures in one or more populations would be expected to produce large frequency differences between populations (i.e., high Fst values). To explore this possibility, we calculated Fst [28] for all frequency-matched SNP pairs versus frequency-matched and perfectly correlated SNPs. At the short distances (i.e., 0 to 50 kb), SNPs that are perfectly correlated revealed similar average Fst values compared to frequency-matched SNPs. However, at longer distances (greater than 150 kb), perfectly correlated SNPs show significantly (*p* < 0.05) higher Fst values (Figure 5), consistent with natural selection.

## Discussion

In this report, we explore the decay of LD as a function of physical distance and SNP allele frequency. Our results show that allele frequency matching between SNP pairs, or minimizing the allele frequency difference between SNPs, provides a more sensitive and useful metric for analyzing LD across the human genome. Although an entirely frequency-independent measure of LD is not possible [16], frequency matching between SNP pairs reduces the influence of frequency when calculating pairwise *r* ^2^ values. Frequency-matched SNPs revealed a significant increase in the extent of LD in genic versus intergenic regions. This increase could not be explained by differences in the recombination rates between these regions. By examining SNPs in perfect LD, we observed an excess in the proportion of perfectly correlated, high frequency, derived alleles in genic regions, providing suggestive evidence for natural selection as well as the observed elevated LD.

When assessing LD, the metric D′ is often used because it is recombination based, and LD between nearby SNPs approaches 1 independent of allele frequency (e.g., [15,29]. D′ is defined to be maximal (D′ = 1) when there is no evidence for historical recombination (i.e., at most three of the four possible haplotypes are observed). Compared to D′, *r* ^2^ is always lower and the average *r* ^2^ value is usually much less than 1 even for closely spaced markers. However, the standard *r* ^2^ metric does appear to perform better at unlinked markers because it approaches 0, unlike D′ which has a significant offset due to its frequency dependence [17] (Figure S6A). For this dataset, the average D′ values are very high (>0.5) for low-frequency (0.1 < MAF < 0.2), unlinked SNP pairs, due to the small sample size in this study (46 to 48 chromosomes). This effect can be greatly reduced by normalizing by the expected range (e.g., [14]), which produces a greater range of D′ values and reveals little difference across SNPs of different frequencies (Figure S6B). Using frequency matching, we are able to reduce the frequency dependence of *r* ^2^ and produce values that spanned almost the entire theoretical range from 0 to 1 (Figure 1). Furthermore, with this matching we observe a decay in LD that depends only on factors such as population history, mutation, recombination, and, possibly, gene conversion rates [10]. It is interesting to note that the average LD decay curves calculated using D′ normalized according to the background level are similar to the frequency-matched curves calculated using *r* ^2^.

Contrary to previous studies [24–26], the average LD curves are similar for both low- and high-frequency SNPs when SNPs are frequency matched (Figure 2A). Yet, we do observe larger LD blocks for lower-frequency common SNPs (10% to 20% MAF) compared to high-frequency SNPs (40% to 50% MAF, Figure 2B). This disparity in average LD block size and average LD decay is likely due to the overlapping and interleaved characteristic of perfectly correlated, low-frequency blocks. When compared to high-frequency SNP blocks, low-frequency blocks are more likely to overlap each other (unpublished data), and the correlations between frequency-matched SNPs that reside in different blocks are low. Even though low-frequency blocks of common SNPs are longer, the average LD between low-frequency SNPs is weighted based on its association within the local block structure. SNP pairs that belong to the same block lead to high LD, whereas SNP pairs that span blocks show lower LD. To illustrate this effect, we calculated the number of tagSNPs required to ascertain all the SNPs at *r* ^2^ > 0.8 in the European sample using a greedy algorithm [5]. We find that approximately 30% more tagSNPs are needed to capture all of the lower-frequency common SNPs (10% to 20% MAF, 86,591 tagSNPs) compared to the high-frequency common SNPs (40% to 50% MAF, 62,907 tagSNPs). Because more low-frequency SNPs are present in the genome (278,851 at frequencies between 10% and 20% versus 206,755 for SNPs at frequencies between 40% and 50%), approximately one tagSNP captures the same number of SNPs (approximately 3.2 SNPs) independent of frequency. Overall, low-frequency common SNPs are not surrounded by large regions of LD and will not lead to drastic reductions in the number of SNPs needed for association studies.

Recent studies have indirectly suggested that genic regions have higher levels of LD than intergenic regions, and it has been reported that recombination occurs preferentially outside of genes on human chromosomes 19 and 22 [30]. Additionally, as previously reported [1], fewer tagSNPs will be needed to capture genetic associations in genic versus nongenic regions, which suggests the action of selective forces within genes. Using the same dataset, we examined the fraction of perfectly correlated SNP pairs to confirm and quantitate LD differences in all three populations (Figure 3). Overall, genic regions showed a significantly larger fraction of perfectly correlated SNPs compared to intergenic regions at physical distances between 20 and 300 kb in all samples and up to 400 kb in the European and Han Chinese samples. This trend was still evident after correcting for large-scale structural variations that may introduce LD artifacts (see Materials and Methods) and was not due to baseline differential recombination rates in genic versus intergenic regions (Figure S3). While the average recombination rate was the same and independent of genomic context, the recombination rates for the perfectly correlated SNPs were in fact higher in genic regions (Figure S4). This would predict that extended LD would occur in intergenic regions compared to genic regions, contrary to our observations.

The mechanism for the higher levels of LD in genic regions is unclear but was not based on potential biases due to recombination rates. However, we do find that perfectly correlated SNP pairs are more likely to have a high frequency (>50%) derived allele at both pair positions compared to SNP pairs that were not perfectly correlated. This excess of high-frequency–derived alleles (among perfectly correlated SNPs) is unlikely under a neutral model at extended physical distances (Figure S5). This is illustrated by coalescent simulations which show that beyond approximately 200 kb only a very small fraction (less than 3%) of the perfectly correlated SNP pairs would have both derived alleles greater than 50% (Figure S5). In perfectly correlated SNP pairs, this elevated fraction of high-frequency–derived alleles is consistent with selective pressures that have driven a fraction of the SNPs to high frequencies, while surrounding SNPs also become high frequency by hitchhiking [20]. In addition to driving surrounding SNPs to high frequencies, hitchhiking will also maintain high levels of LD between the SNPs. Another signature of population-specific selection is a rapid rise in allele frequency for an SNP under selection, potentially leading to increased differences in allele frequencies between populations. Our analysis revealed that SNPs that are correlated over longer distances have significantly higher than expected average Fst values (Figure 5), which suggests independent selective forces acting on these SNPs in each population.

Various tests based on measures such as nucleotide diversity or population structure (e.g., [31–33]) can be used to search the human genome for signatures of selection. Recently, several studies used the Perlegen and HapMap datasets to search for signatures of selection using nucleotide diversity, or similar, measures [21,34,35]. While these studies have concentrated on SNP diversity, LD-based measures may also be used to search for signatures of positive selection [18–20,36]. Our results confirm that LD-based approaches can be used to identify the most extreme regions and will primarily have power to detect active selection prior to allelic fixation or selection that has acted on standing genetic variation [37].

Estimates for the number of genes contributing to the excess LD in genic regions reveal that less than approximately 3% of the genes in the genome (i.e., less than 600) are contributing to the excess LD in genic regions (i.e., removing the 3% of the genic regions that contribute the most excess LD minimizes the differences in LD between genic and intergenic regions) (Table S1). This estimate of the fraction of genes under selection is based on our empirical results and does not represent extremes of a “selection metric” distribution as other recent whole-genome studies have used (e.g., [18,34,35]). However, our LD approach suggests that considering the approximately 3% extremes of the distribution is a reasonable approach when searching for regions of the human genome under selection. We show that the frequency dependence of *r* ^2^ can be minimized by frequency-matching SNPs. It should be noted that this number (3%) is a rough estimate and that some “selected” genes may not be identified.

Practically, our observations have applications for single-marker association studies where markers that have similar frequencies to the causative SNP can have high correlations with the causative allele. Indirectly, this property of *r* ^2^ has been previously observed, because larger sample sizes are required for mapping when an SNP has a very different frequency to that of the causative polymorphism [38]. For low-penetrance phenotypes or low-frequency risk alleles, where the overall power to detect risk alleles, even when they are directly genotyped, is already low, allele frequency matching will be especially important. It is possible that this frequency-matching problem could be reduced further by performing multimarker associations that increase “marker” frequencies and increase the likelihood of tagging the same branch as the risk allele (e.g., [3,39,40]).

## Materials and Methods

### Calculating LD.

For all pairs of SNPs with alleles *A/a* and *B/b* and corresponding fixed frequencies *p_A_*, *p_a_*, *p_B_*, and *p_b_* in a particular sample, we can form a two-by-two matrix that describes the haplotypes made by the two SNPs:

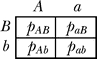
where *p*_ij_ is the frequency of the ij haplotype. The sum of the haplotype columns or rows is equal to the frequency of the allele common to the haplotype (i.e., *p_AB_* + *p_Ab_* = *p_A_*) and all four haplotype frequencies sum to 1. Using these definitions, LD between SNP pairs using the metric *r* ^2^ is given by the equation:

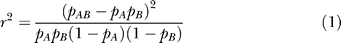
where *p_A_* is the frequency of the minor allele at the first SNP and *p_B_* is the frequency of the minor allele at the second SNP. The metric *r* ^2^ varies between 0 and 1 where 0 means that the SNPs are completely uncorrelated and 1 means that the two SNPs are perfectly correlated. To calculate Equation 1, the unknown haplotypes in individuals that are heterozygous for both SNPs are estimated using the expectation-maximization (EM) algorithm [41]. Working with simulated data from coalescent models, we have examined the accuracy of estimating haplotypes using this method. For the sample sizes used in this study, the average LD is biased upward slightly but consistently: average *r* ^2^ values are 0.0015 to 0.016 higher for the estimated haplotypes compared with the actual haplotypes.

In the absence of recombination, Equation 1 can be further simplified to estimate the maximum expected *r* ^2^ value according to the allele frequency spectrum of the data. When calculating LD, we are free to arbitrarily define the alleles when creating the two-by-two matrix, so we have chosen *p_A_* and *p_B_* as the minor alleles such that *p_A_* ≤ *p_B_* ≤ 0.5. For this scenario, in the absence of recombination, only two possible values exist for *r* ^2^ depending on whether the *A* allele occurs as a subset of the *B* lineage (i.e., *p_AB_* = *p_A_*) or as a subset of the *b* lineage (i.e., *p_AB_* = 0) (Figure S7). The maximum possible *r* ^2^ value occurs when the *A* allele is a subset of the *B* lineage and *p_AB_* is equal to *p_A_*. Under this scenario, the maximum *r* ^2^ value occurs and Equation 1 simplifies to:

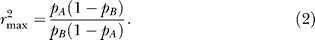

Equation 2 shows that the maximum *r* ^2^ value is always less than unity unless *p_A_* = *p_B_*. When the *A* allele is a subset of the *b* lineage, *p*_AB_ = 0 and the minimum *r* ^2^ value occurs. For this scenario Equation 1 simplifies to:

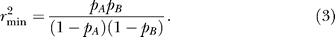
In the absence of recombination where only three of the four possible haplotypes are possible, Equations 2 and 3 give the only possible values for *r* ^2^ for allele frequencies *p_A_* and *p_B_*.

Because, in the absence of any historical recombination events between the two SNPs, only two *r* ^2^ values are possible for a particular pair of SNPs with MAFs *p_A_* and *p_B_*, the expected LD will be a function of the fraction of SNPs that fall into each possible scenario. The expected LD in the absence of recombination will be a function of the allele frequencies and is given by:

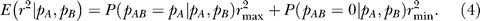



We estimated the probabilities in Equation 4 by simulating 2,000 nonrecombinant blocks of length of 500 kb using the coalescent [42]. At high frequencies this value will be greater both because 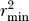 will be larger and the probability of having the minimal value will also be smaller. We estimated the probability of observing each *r* ^2^ value (in the absence of recombination) using coalescent simulations and then calculated the expected maximum values for all possible pairs of SNP frequencies (Figures 2 and S8). Combining this information with the SNP frequency spectrum of the data, we estimated the maximum expected *r* ^2^ values for the data (dashed blue lines in Figure 1).

It is interesting to note that when *p_A_* ≠ *p_B_*, the probabilities of the *A* allele occurring within the *B* allele subtree is approximately *p_B_* (Figure S8C). By putting this value into Equation 4, the expected LD simplifies to:

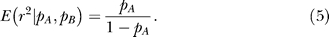
The predicted LD from coalescent modeling agrees quite well with this estimate when the minor allele frequencies are different (Figures S8D and S9). When the frequencies are not matched, the maximum, nonrecombinant *r* ^2^ value is only dependent upon the frequency of the rarer of the two minor alleles. Conversely, when the frequencies *p_A_* and *p_B_* are equal, then in general *P*(*p_AB_* = *p_A_*) ≫ *p_B_* and Equation 5 is no longer a good estimate of *r* ^2^ (e.g., Figures S8 and S9).

To calculate the fraction of SNP pairs in perfect LD (*r* ^2^ = 1), we compared genotypes rather than estimating haplotypes. To do this, we first removed all SNPs with any missing genotype information. Removing all SNPs with missing genotype information reduced our usable number of SNPs from 1,242,434 to 1,027,752 in the European data, from 1,434,265 to 1,083,911 in the African-American data, and from 1,128,206 to 945,832 in the Han Chinese data. For the remaining SNPs, we redefined the genotypes—0 = homozygous for the common allele, 1 = heterozygous, and 2 = homozygous for the rare allele—and performed a string comparison between all SNPs with the same number of minor allele observations. SNPs with the same descriptive string are perfectly correlated for our analysis. Since only SNPs with the same frequencies can be perfectly correlated (e.g., Equation 2), we only compared SNPs with the minor allele observed the same number of occurrences in this analysis. In addition to removing SNPs with missing genotype information, we also removed SNPs where the minor allele has fewer than four occurrences, because rarer SNPs are more likely to be perfectly correlated by chance. Removing the lowest-frequency SNPs limits the problem; unlinked SNPs with the minor allele observed at least four times in a sample of 48 chromosomes have a less than 0.01% chance of being perfectly correlated according to our definition, and higher frequencies are even less likely to be perfectly correlated by chance.

### Genic and intergenic regions.

We separated SNPs into those located in genic regions (intragenic) and to those that fell between genic regions (intergenic) using the UCSC definitions of known genes using human genome build (35). For our analysis, we define a gene as including 5 kb upstream and downstream of the start and stop codons to limit gene effects on the surrounding nongenic regions. Cases where the genes overlap (or are within 10 kb of each other) are combined together to make a single, larger “genic region.” Additionally, we excluded all SNPs that fell within predicted (but not “known”) genes from our analysis since these regions may bias our estimates. For our analysis, there are 10,334 genic regions with at least two SNPs, and an average genic region is approximately 106 kb and contains 60 SNPs. There are 8,502 intergenic regions with at least two SNPs, and an average intergenic region is approximately 211 kb and contains 112 SNPs. When we test whether SNPs are perfectly correlated, we only include SNP pairs where both SNPs are in the same contiguous genic (or intergenic) segment.

Examination of the data to detect regions of the genome that contained the greatest number of perfectly correlated SNPs, revealed a segment of the genome that showed unusual LD characteristics due to large structural variation, an approximately 1-Mb inversion located on Chromosome 17 [43]. This region contributed a large fraction of the perfectly correlated SNPs, accounting for 2.6% (approximately 22,000) of all perfectly correlated genic SNPs in the Europeans and 4.3% (approximately 15,000) of all perfectly correlated genic SNPs in the Africans. To ensure that other undiscovered genomic structural variations were not driving the genic/intergenic differences shown in Figure 2, we determined the regions with the largest number of perfectly correlated SNPs and repeated our analysis without these regions. Specifically, we separated the genome into 500-kb segments and removed the 100 regions (50 Mb, 1.7% of the genome) that contributed the most perfectly correlated SNP pairs. This led to the removal of 39,126 (1.9%) of the perfectly correlated SNP pairs for the Europeans, 21,486 (2.6%) of the perfectly correlated SNP pairs for the Africans, and 21,721 (1%) of the perfectly correlated SNP pairs for the Han Chinese. Removal of this data only resulted in a slight shift of the genic LD curve for the both the Europeans and African-Americans and essentially no shift in the LD curve for the Han Chinese which does not carry the large inversion on Chromosome 17 [43].

### Recombination rates and ancestral alleles.

We assessed recombination across all populations and between SNP pairs using the recombination rates estimated from the deCode dataset [44]. To estimate the recombination rate between any two SNPs, we assumed that recombination rates varied linearly with physical distance between the estimates of the deCode dataset. We then integrated between each SNP pair to get the average recombination rate for that SNP pair where the recombination rate at each base position is calculated by linearly interpolating from the surrounding values from the deCode data.

We determined the derived allele of each SNP by comparing the chimpanzee reference sequence to the human reference sequence (hg17) using the chimpanzee-human pairwise alignments available from the UCSC Genome Browser (http://hgdownload.cse.ucsc.edu/goldenPath/panTro1/vsHg17). Of the 1,539,287 SNPs with genotype data on Chromosomes 1 through 22, we were not able to get a chimpanzee allele for 50,718 (3.3%). Of the remaining SNPs, 14,447 (1%) were inconsistent with the chimpanzee allele and 1,409 (0.1%) were inconsistent with the human allele. Of the remaining 1,472,713 SNPs, both alleles were the reverse complement of each other at 224,317 (15%) sites. The approximately 1% of the chimp alleles that did not agree with either allele of our human SNPs is consistent with the approximately 1.06% fixed divergence between chimps and humans [45]. Additional errors in the called ancestral allele will occur when there is a fixed difference between the species but the human polymorphism mutates to the chimpanzee allele, or the chimpanzee reference sequence contains a chimpanzee mutation from the ancestral allele. These errors will be undetectable but should only occur half as often as the fixed differences, or approximately 0.5% of our ancestral alleles should be miscalled. Errors of this magnitude will not change our results.

## Supporting Information

Figure S1 LD with Physical Distance between SNP Pairs for Frequency Bins 0.1 to 0.2 (Red), 0.2 to 0.3 (Green), 0.3 to 0.4 (Blue), and 0.4 to 0.5 (Violet) The frequency bins for SNP pairs in (A) African-American and (B) Han Chinese samples.
(483 KB PDF)

Figure S2 Extent of Blocks of Perfect LD in the African-American and Han Chinese Populations as a Function of Minor Allele Frequency (A) African-American populations.
(B) Han Chinese populations.
Curves represent the 0.05 (bottom), 0.50 (middle), and 0.95 (top) quantiles of the distributions.
(464 KB PDF)

Figure S3 Average Recombination Rate for the SNP Pairs Used to Calculate the Curves of Perfectly Correlated SNP Pairs Shown in Figure 1
Average recombination rate for the SNP pairs used to calculate the curves of perfectly correlated SNP pairs shown in Figure 1 for the European (A), African-American (B), and Han Chinese (C) data. Red curves are for the intergenic regions, and blue curves are for the genic regions. Recombination rates are estimated as described in Materials and Methods.
(456 KB PDF)

Figure S4 Average Recombination Rate for All of the Perfectly Linked SNP Pairs in Figure 3
The blue lines are for the genic SNPs, and the red lines are for the intergenic SNPs in the European (A), African-American (B), and Han Chinese (C) data.
(472 KB PDF)

Figure S5 Fraction of High-Frequency, Perfectly Correlated SNP Pairs and Extent of LD within This Dataset and Theoretical Models (A) Fraction of perfectly correlated (*r* ^2^ = 1) SNP pairs where both derived alleles are the more common allele from coalescent simulations (solid line). Dashed line shows the fraction of imperfectly correlated (*r* ^2^ < 1) SNP pairs where both derived alleles are the more common allele.
(A′) Fraction of all SNP pairs that are perfectly correlated by distance for the coalescent simulations from (A).
Results from the data are shown for the genic (blue) and intergenic (red) regions for the European (B and B′), African-American (C and C′), and Han Chinese (D and D′) samples.
(539 KB PDF)

Figure S6 Decay of LD Calculated by D′ with Physical Distance for SNP Pairs of Frequency 0.1 to 0.2 (Red), 0.2 to 0.3 (Green), 0.3 to 0.4 (Blue), and 0.4 to 0.5 (Violet) for the European Samples (A) LD decay calculated using all possible frequency comparisons. Dashed lines show the theoretical expected minimum based on allele frequencies.
(B) The LD decay curves from the top figure normalized by the minimum expected LD values (shown as dashed lines in the top figure) for the frequency distribution [i.e., D_norm_ = (D′ − D_min_)/(1 − D_min_)].
(492 KB PDF)

Figure S7 Illustration of the Possible Haplotypes that Can Occur in the Absence of Recombination Without recombination, if p_B_ has the highest frequency of all three minor alleles, then the other two minor alleles can only occur either on the *B* branch or the non-*B* branch. If SNPs occur on the *B* branch like the *a* → *A* mutation, then it will have the maximum possible LD value with the *b/B* SNP. If the mutation occurs on the non-*B* branch as the *c* → *C* mutation does, then it will have the minimum LD value.
(443 KB PDF)

Figure S8 Theoretically Expected Linkage Disequilibrium without Historical Recombination Theoretical maximum (A) and minimum (B) *r* ^2^ values in the absence of recombination for all possible minor allele frequencies f_B_ and f_A_ (where f_A_ < f_B_ < 0.5) calculated using Equations 2 and 3 (see Materials and Methods).
(C) Expected fraction of SNP pairs with the maximum possible LD occurs in the absence of recombination as a function of the minor allele frequencies f_A_ and f_B_. LD values are estimated from 20,000 coalescent simulations of 100 chromosomes of length 100 kb.
(D) Expected LD between SNPs without recombination as a function of the minor allele frequencies f_A_ and f_B_. Expected values are calculated using the estimates from the results shown in (A) through (C) (see Materials and Methods).
(707 KB PDF)

Figure S9 Difference between LD Values in the Simulations without Recombination and the Expected Values from Equation 5
Simulations are described in the caption to Figure S8.
(A) Percent difference in *r* ^2^ between the expectations and simulations.
(B) Actual differences in *r* ^2^ between the expected values and the simulated values.
(544 KB PDF)

Table S1 The Top 3% of Genes that Contribute the Most to the Excess LD in Genes (47 KB XLS)

## Abbreviations

LD: linkage disequilibrium
MAF: minor allele frequency
SNP: single nucleotide polymorphism
