# Supplementary material for: Allele Frequency Matching Between SNPs Reveals an Excess of Linkage Disequilibrium in Genic Regions of the Human Genome
Source: PLoS Genet. 2006 Sep 8;2(9):e142. doi: 10.1371/journal.pgen.0020142 (PMC1560400; doi:10.1371/journal.pgen.0020142)
Supplement: Supplementary file 2 [file pgen.0020142.sg002.pdf]

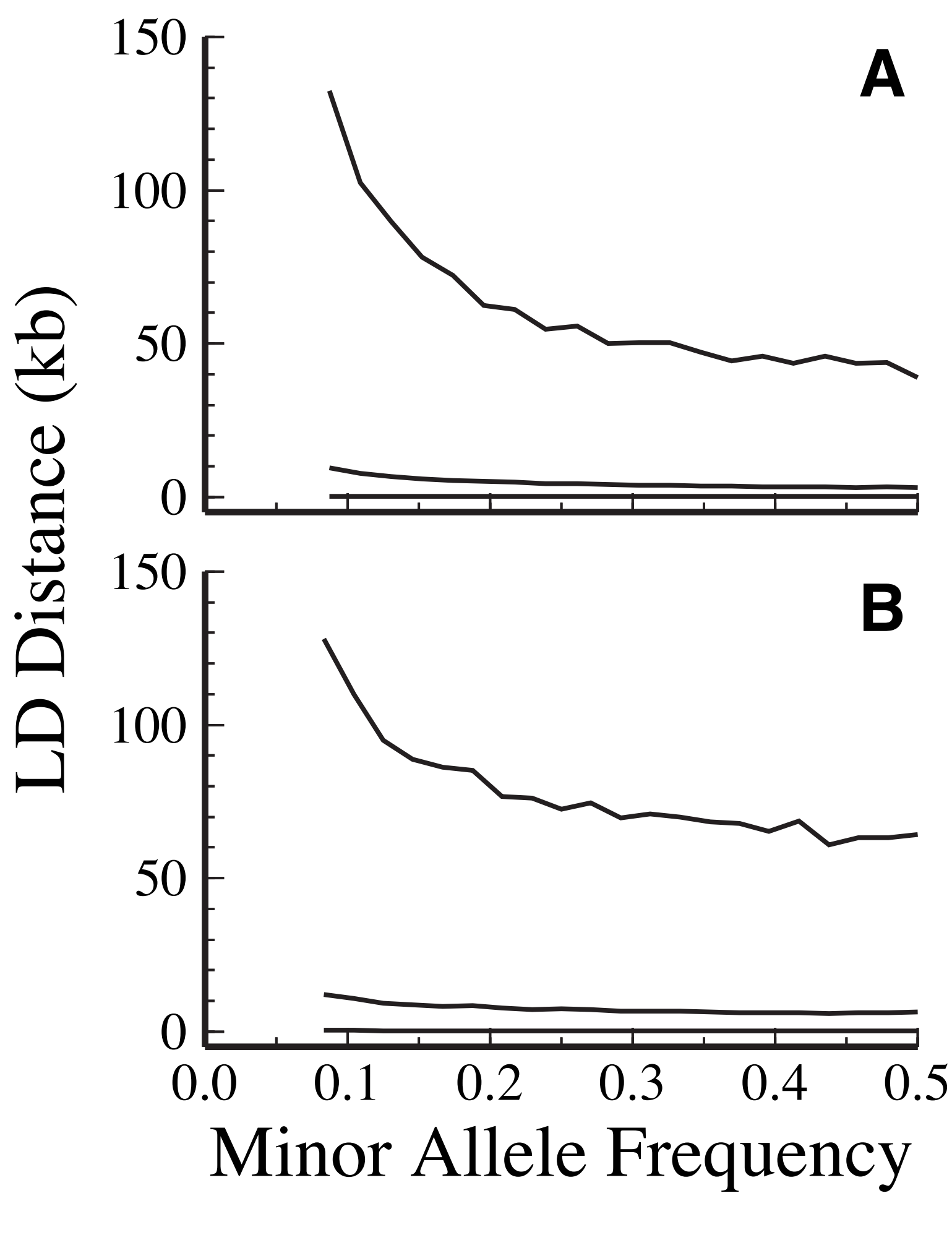

A
B
150
100
50
0
LD Distance (kb)
0.0
0.1
0.2
0.3
0.4
0.5
Minor Allele Frequency
