# Supplementary material for: Allele Frequency Matching Between SNPs Reveals an Excess of Linkage Disequilibrium in Genic Regions of the Human Genome
Source: PLoS Genet. 2006 Sep 8;2(9):e142. doi: 10.1371/journal.pgen.0020142 (PMC1560400; doi:10.1371/journal.pgen.0020142)
Supplement: Supplementary file 3 [file pgen.0020142.sg003.pdf]

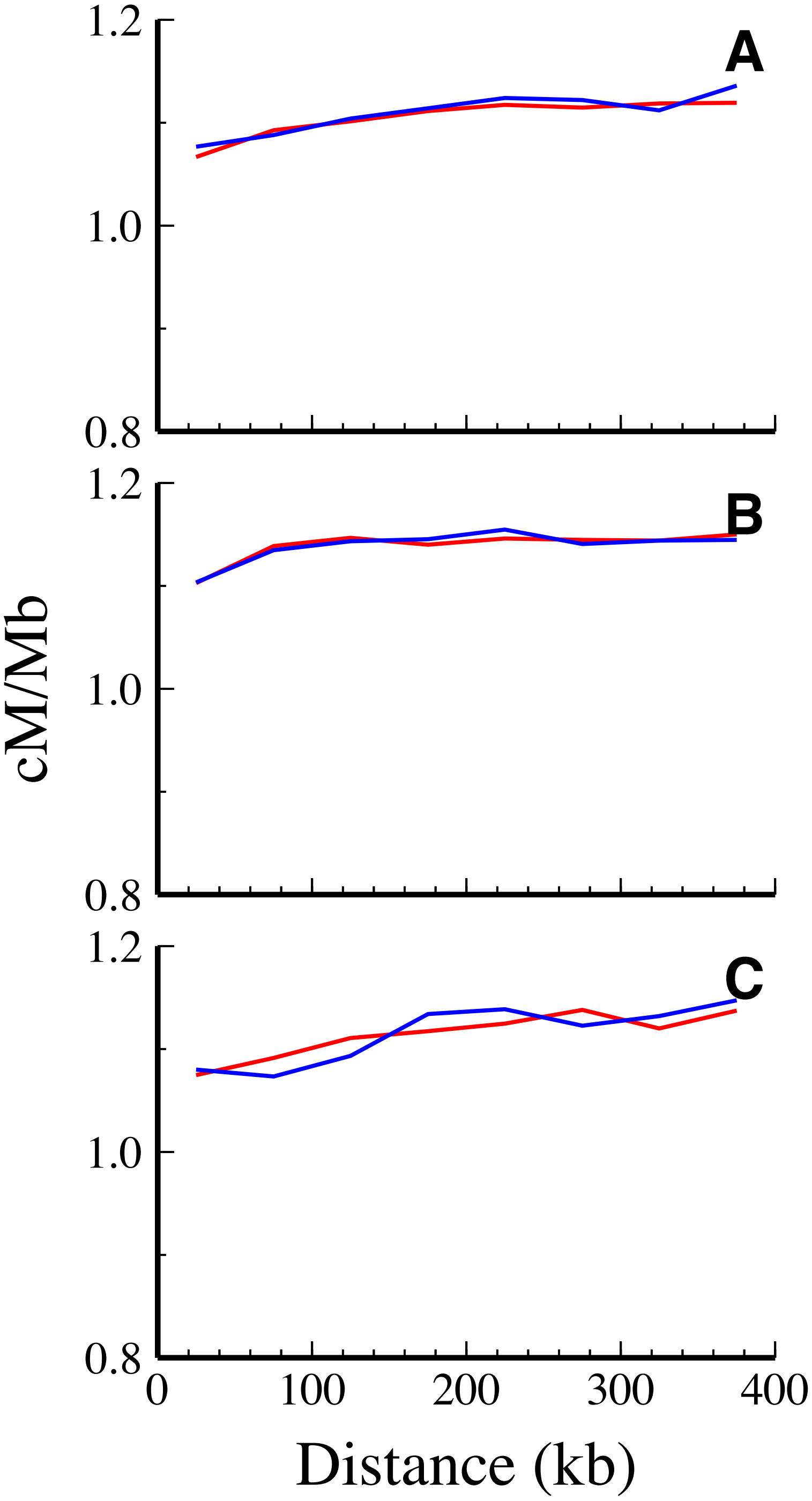

1.2
1.0
0.8
A
B
C
cM/Mb
0
100
200
300
400
Distance (kb)
