# Supplementary material for: Allele Frequency Matching Between SNPs Reveals an Excess of Linkage Disequilibrium in Genic Regions of the Human Genome
Source: PLoS Genet. 2006 Sep 8;2(9):e142. doi: 10.1371/journal.pgen.0020142 (PMC1560400; doi:10.1371/journal.pgen.0020142)
Supplement: Supplementary file 5 [file pgen.0020142.sg005.pdf]

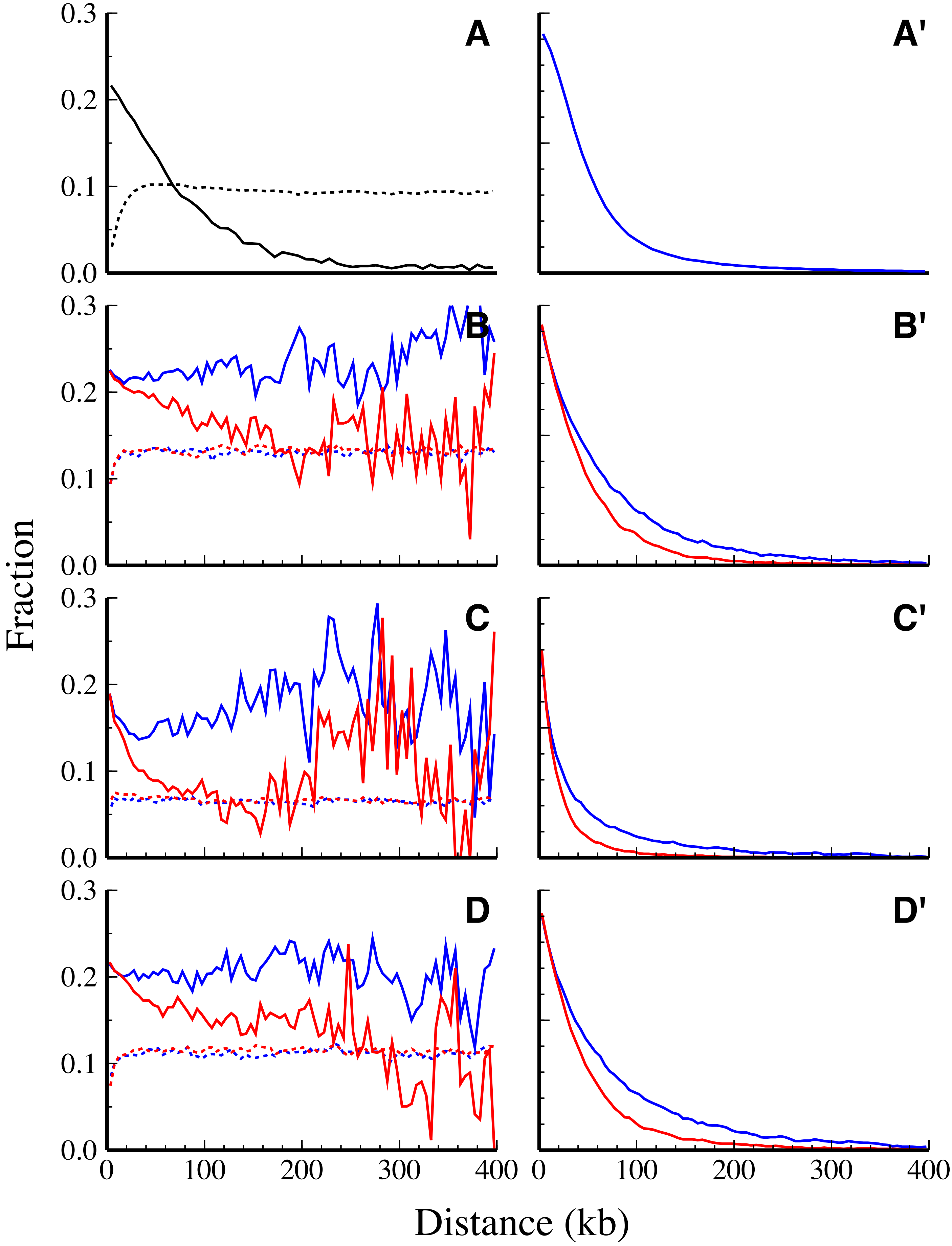

A
A'
B
B'
C
C'
D
D'
0.3
0.2
0.1
0.0
0
100
200
300
400
Fraction
Distance (kb)
