# Supplementary material for: Allele Frequency Matching Between SNPs Reveals an Excess of Linkage Disequilibrium in Genic Regions of the Human Genome
Source: PLoS Genet. 2006 Sep 8;2(9):e142. doi: 10.1371/journal.pgen.0020142 (PMC1560400; doi:10.1371/journal.pgen.0020142)
Supplement: Supplementary file 6 [file pgen.0020142.sg006.pdf]

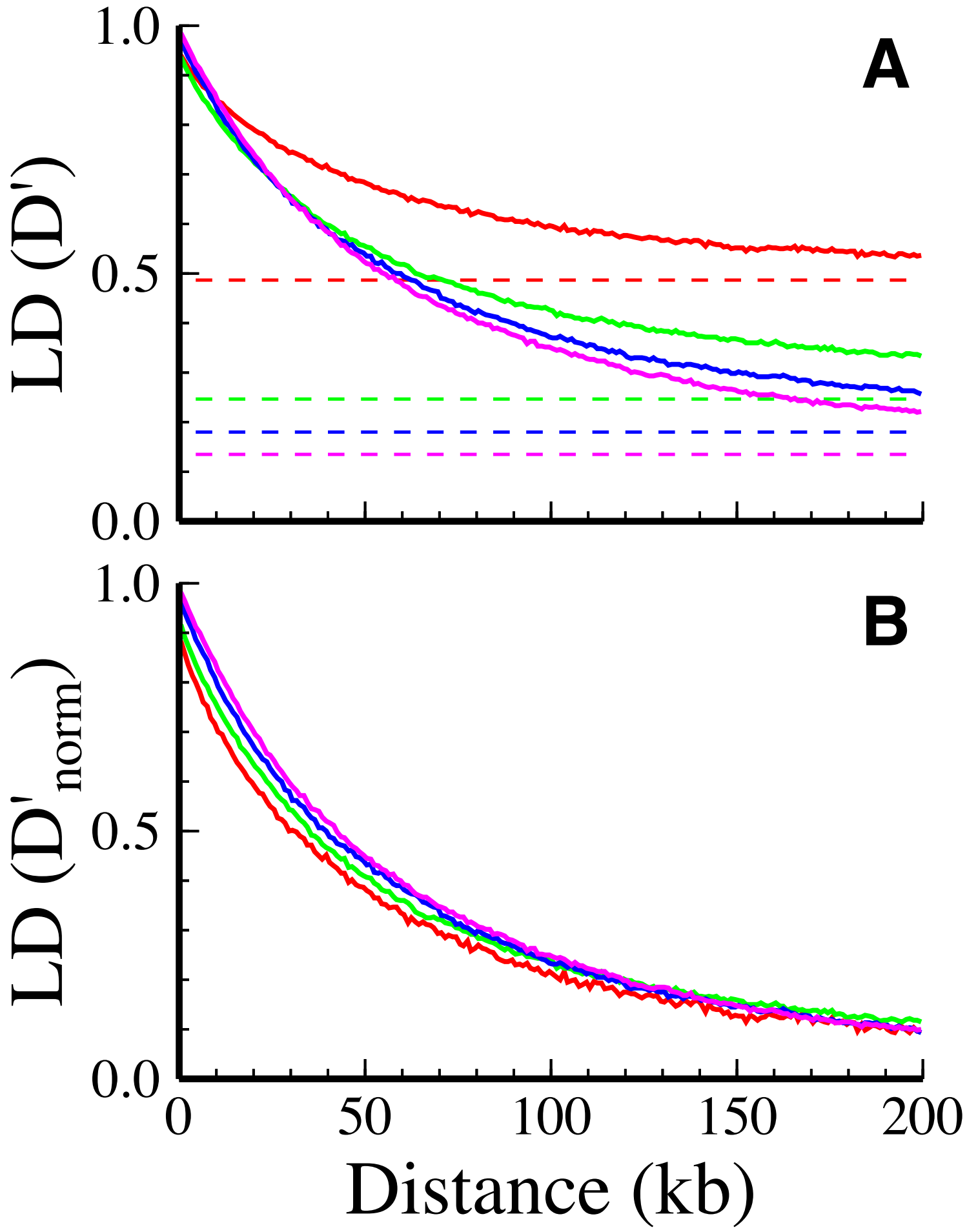
A
B
LD (D')
LD (D'norm)
1.0
0.5
0.0
0
50
100
150
200
Distance (kb)
