# Supplementary material for: Allele Frequency Matching Between SNPs Reveals an Excess of Linkage Disequilibrium in Genic Regions of the Human Genome
Source: PLoS Genet. 2006 Sep 8;2(9):e142. doi: 10.1371/journal.pgen.0020142 (PMC1560400; doi:10.1371/journal.pgen.0020142)
Supplement: Supplementary file 7 [file pgen.0020142.sg007.pdf]

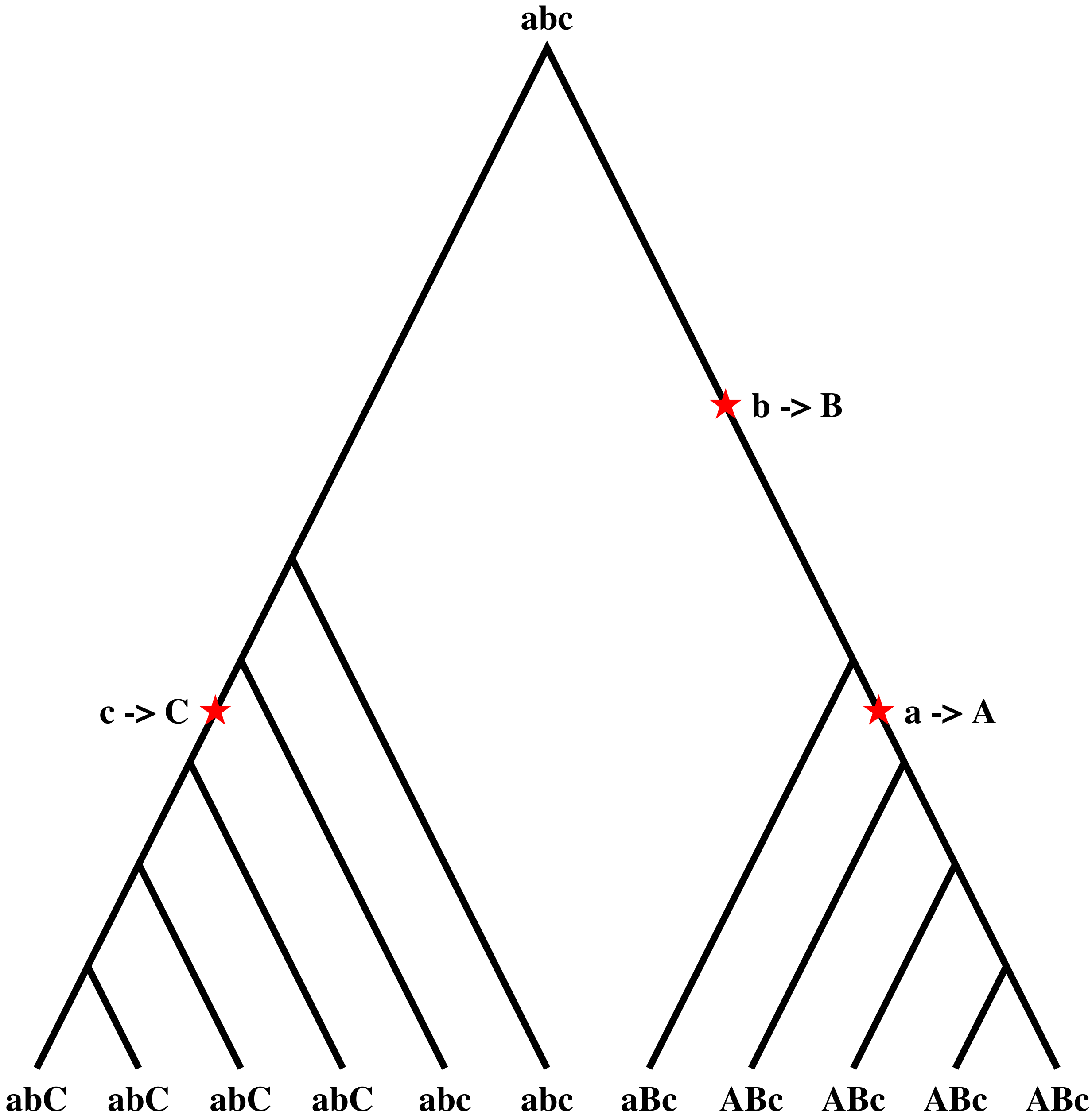
abc
b -> B
c -> C
a -> A
abC
abC
abC
abC
abc
abc
aBc
ABc
ABc
ABc
ABc
