# Supplementary material for: Allele Frequency Matching Between SNPs Reveals an Excess of Linkage Disequilibrium in Genic Regions of the Human Genome
Source: PLoS Genet. 2006 Sep 8;2(9):e142. doi: 10.1371/journal.pgen.0020142 (PMC1560400; doi:10.1371/journal.pgen.0020142)
Supplement: Supplementary file 8 [file pgen.0020142.sg008.pdf]

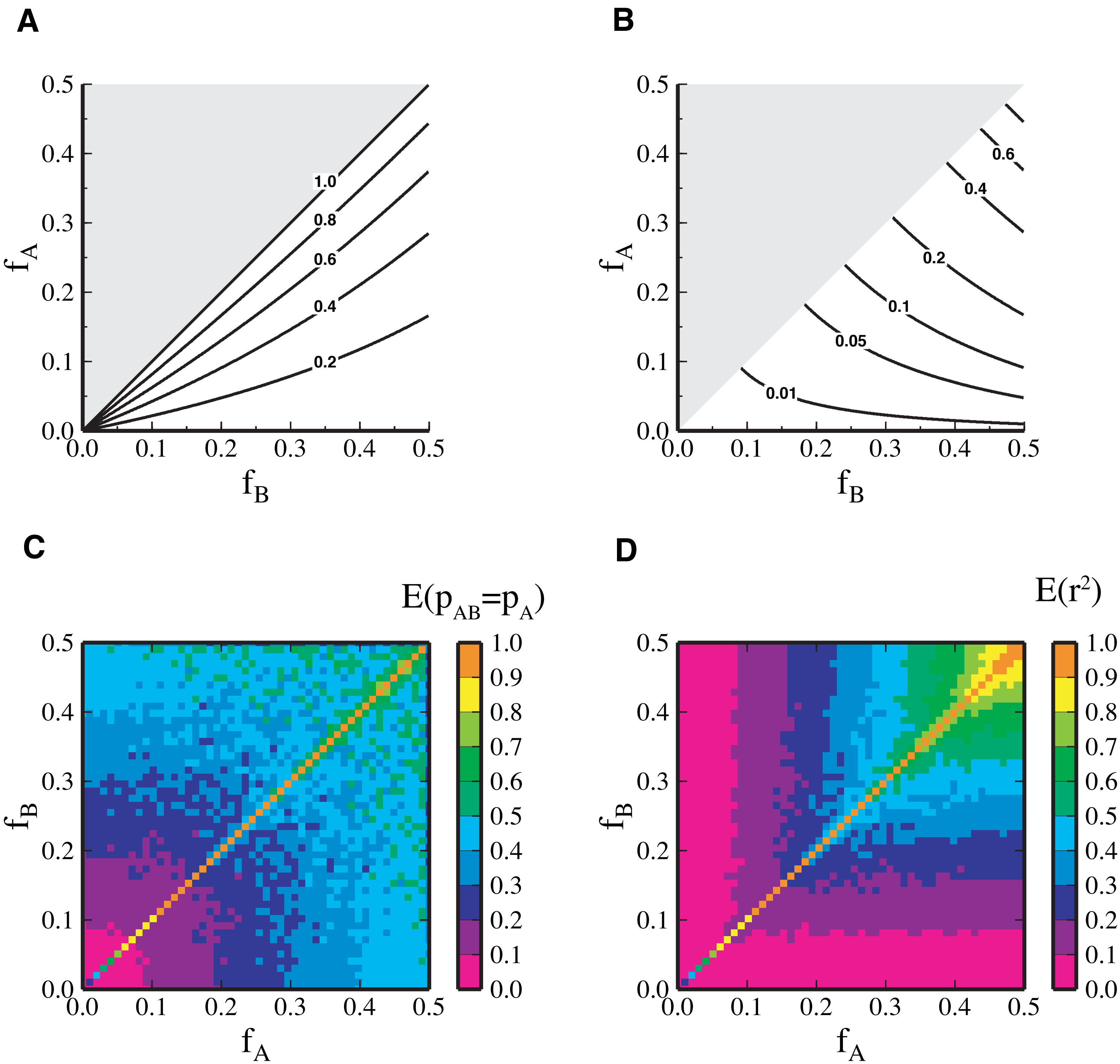

A
B
C
D
f_A
f_B
1.0
0.8
0.6
0.4
0.2
0.01
0.05
0.1
$E(p_{AB}=p_A)$
$E(r^2)$
0.0
0.1
0.2
0.3
0.4
0.5
0.6
0.7
0.8
0.9
