# Supplementary material for: Allele Frequency Matching Between SNPs Reveals an Excess of Linkage Disequilibrium in Genic Regions of the Human Genome
Source: PLoS Genet. 2006 Sep 8;2(9):e142. doi: 10.1371/journal.pgen.0020142 (PMC1560400; doi:10.1371/journal.pgen.0020142)
Supplement: Supplementary file 9 [file pgen.0020142.sg009.pdf]

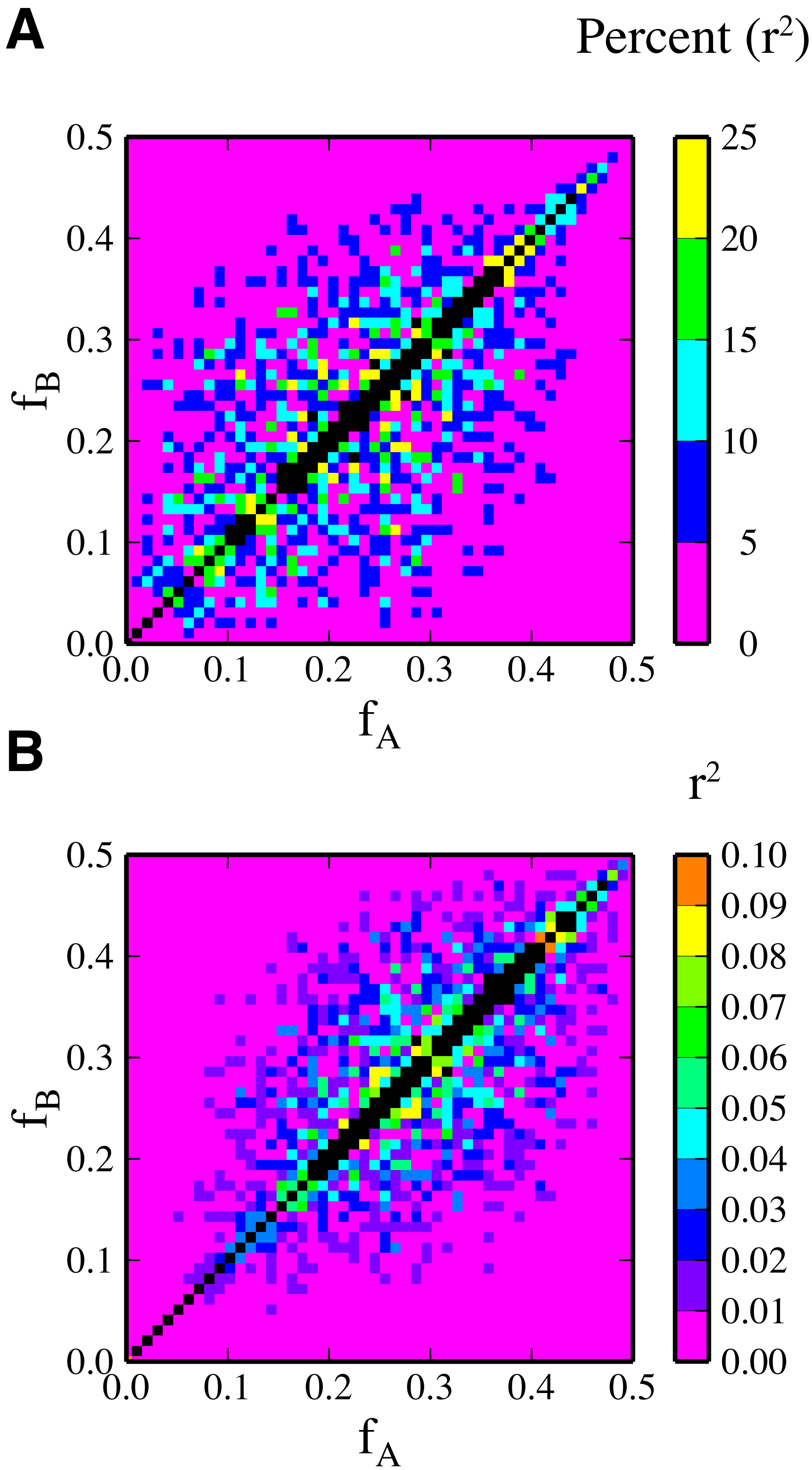

A
Percent (r^2)
0.0
0.1
0.2
0.3
0.4
0.5
f_B
f_A
0
5
10
15
20
25
B
r^2
0.00
0.01
0.02
0.03
0.04
0.05
0.06
0.07
0.08
0.09
0.10
